# Advances in Ca_V_1.1 gating: New insights into permeation and voltage-sensing mechanisms

**DOI:** 10.1080/19336950.2023.2167569

**Published:** 2023-01-15

**Authors:** Hugo Bibollet, Audra Kramer, Roger A. Bannister, Erick O. Hernández-Ochoa

**Affiliations:** a Department of Biochemistry and Molecular Biology, University of Maryland School of Medicine, Baltimore, MD, USA; b Department of Pathology, University of Maryland School of Medicine, Baltimore, MD, USA

**Keywords:** Skeletal muscle, Ca_V_1.1, dihydropyridine receptor (DHPR), calcium channel, voltage-sensor, excitation-contraction (EC) coupling, voltage-clamp, functional site-directed fluorometry

## Abstract

The Ca_V_1.1 voltage-gated Ca^2+^ channel carries L-type Ca^2+^ current and is the voltage-sensor for excitation-contraction (EC) coupling in skeletal muscle. Significant breakthroughs in the EC coupling field have often been close on the heels of technological advancement. In particular, Ca_V_1.1 was the first voltage-gated Ca^2+^ channel to be cloned, the first ion channel to have its gating current measured and the first ion channel to have an effectively null animal model. Though these innovations have provided invaluable information regarding how Ca_V_1.1 detects changes in membrane potential and transmits intra- and inter-molecular signals which cause opening of the channel pore and support Ca^2+^ release from the sarcoplasmic reticulum remain elusive. Here, we review current perspectives on this topic including the recent application of functional site-directed fluorometry.

## Background

**Figure 1.**
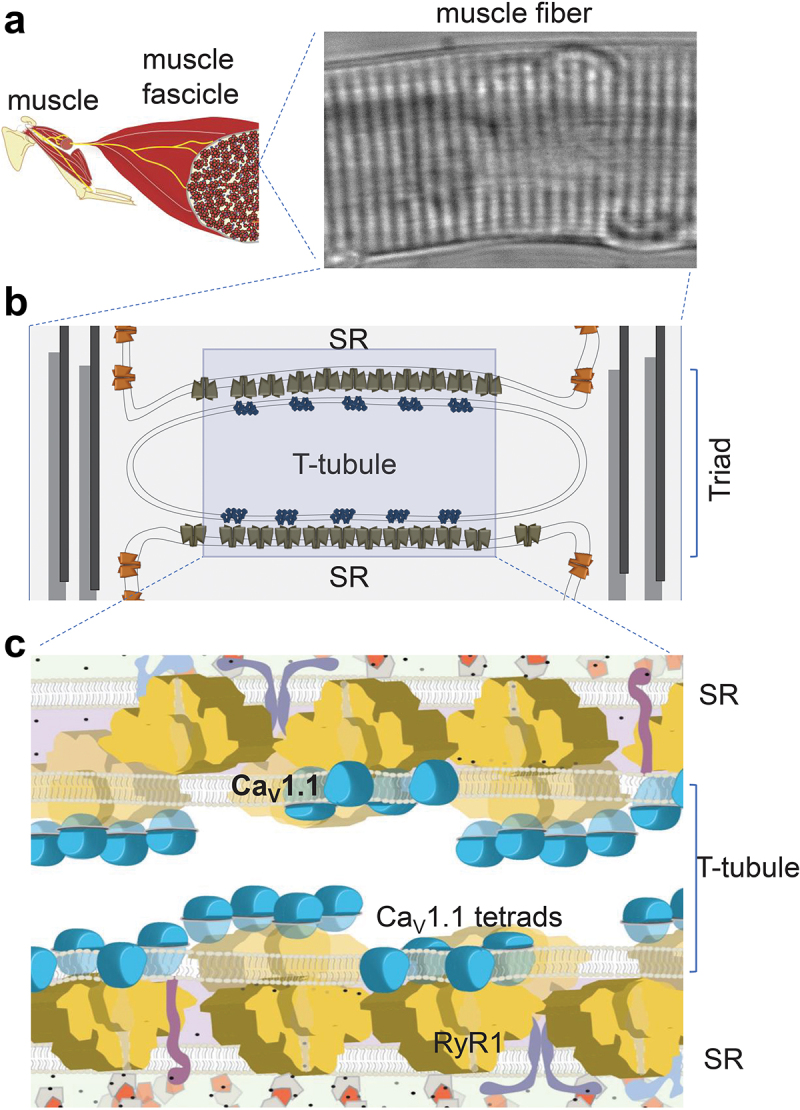
Muscle and Ca_V_1.1 organization. A) Muscle to muscle cell perspective. (*left*) Morphology of a segment of a skeletal muscle fiber (*right*). Note the characteristic striated pattern of muscle fibers, which results from highly organized array between sarcolemma, sarcoplasmic reticulum (SR), contractile elements and cytoarchitecture of the fibers. B) Structure of the triad. The cartoon depicts the triad, a specialized membrane-organelle array formed by the T-tubule and two segments of the terminal junctional SR. The T-tubules are infoldings of the sarcolemma that propagate the action potential radially into the fiber. Ca_V_1.1 (blue) are located at the T-tubules, working primarily as voltage sensors that initiate the early steps of EC coupling. The SR Ca^2+^ release channel, RyR1 (brown), is predominantly located on the junctional domain of the SR surface. Typical profiles of triads (cross-sections) contain only two rows of RyR1 associated with alternating tetrads [2]. C) Detailed architecture of the triad with a focus on Ca_V_1.1 tetrads and RyR1 arrays as shown in (b). About half of the total RyR1s do not associate with Ca_V_1.1, resulting in an alternating pattern of “free” and Ca_V_1.1-associated RyR1s. Note: In addition to Ca_V_1.1 tetrad (blue) and RyR1 (yellow) Ca^2+^ release channels, many other proteins form part of the T-tubule- junctional SR complex (e.g. junctophilin, triadin, junctin, calsequestrin, not indicated here).

The skeletal muscle L-type Ca^2+^ channel (Ca_V_1.1) is the prototypical member of the Ca_V_ family[1]. Ca_V_1.1 has two primary functions: (1) the voltage-sensor for Ca^2+^ release from the sarcoplasmic reticulum (SR) via the type 1 ryanodine receptor (RyR1), and (2) L-type Ca^2+^ channel. Ca_V_1.1 is expressed exclusively in skeletal muscle at triad junctions formed by the plasma membrane of the transverse-tubule network (T-tubules) and the terminal cisternae of the SR (Figure 1a-b). Ca_V_1.1 channels are clustered in groups of four within the plasma membrane of triad junctions. Each group of four Ca_V_1.1 channels is known as a “tetrad” and each tetrad is juxtaposed to one leaf every other quatrefoil RyR1 in the SR membrane forming a checkerboard-like pattern [2–4] (Figure 1c). This unique ultrastructure is a prerequisite for the intermolecular communication between Ca_V_1.1 and RyR1 that supports excitation-contraction (EC) coupling in skeletal muscle [2]. Coupled RyR1 channels mediate rapid Ca^2+^ release from the SR into the cytosol in response to the muscle action potential in the T-tubules, leading to Ca^2+^ binding to troponin C and activation of actin–myosin interactions for muscle contraction. Membrane depolarization of the sarcolemma and T-tubule system also activates Ca^2+^ influx through Ca_V_1.1 [5,6]. While this Ca_V_1.1-dependent Ca^2+^ flux appears to be important for SR refilling during sustained activity [7], it is not necessary for EC coupling in differentiated mature skeletal muscle [8–10].

**Figure 2.**
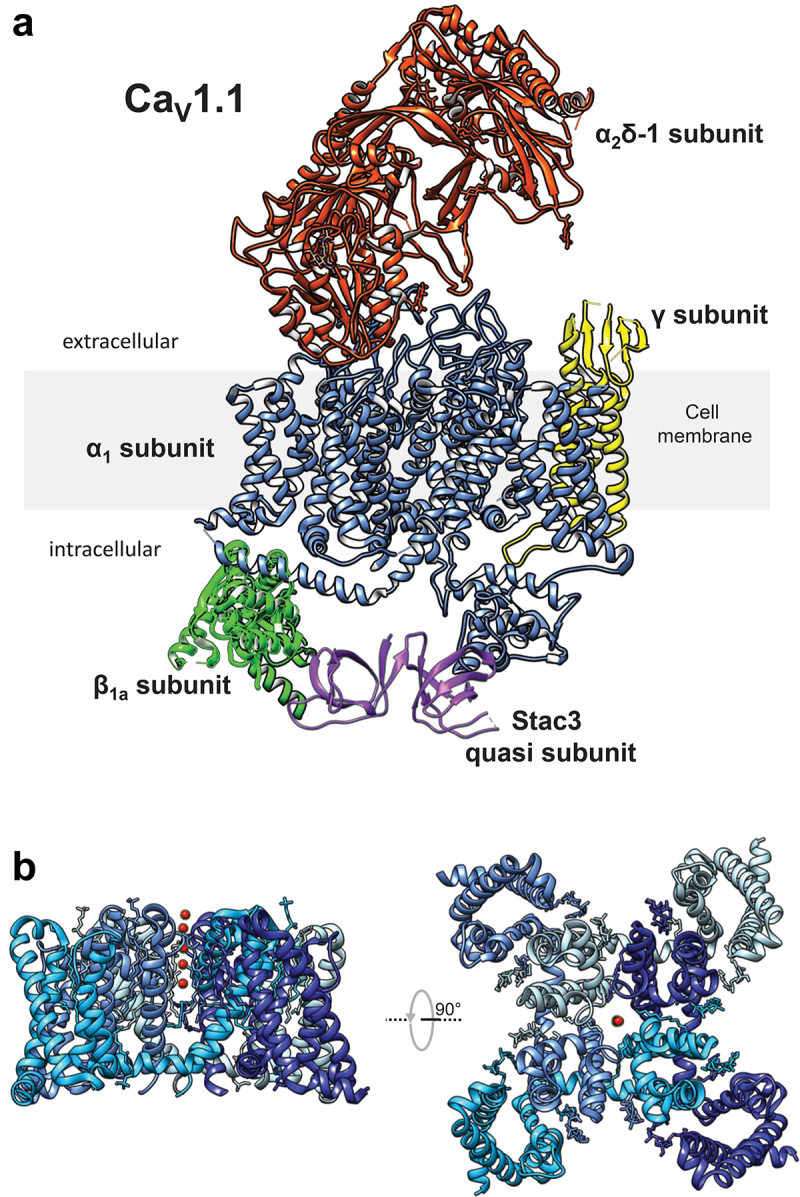
Ca_V_1.1 structure. Heteromultimeric protein complex of Ca_V_1.1. α_1S_, β_1a_, α_2_δ-1, γ subunits, and SH3 domains of Stac3 are colored in blue, green, Orange, yellow, and purple, respectively. B) Side and upper views of the α_1S_ subunit, each domain is shown in shades of blue. Red dots indicate Ca^2+^ ions. Panels A and B were prepared with Chimera [22]. Protein data bank (PDB) IDs: 5GJV (Ca_V_1.1) and 6UY7 (Stac3). STAC3 orientation and position relative to α1 and the β subunits is unknown.

Ca_V_1.1 is a heteromultimeric channel complex formed by a principal α_1S_ subunit [11], auxiliary β_1a_, α_2_δ1, γ_1_ subunits [12–14] and another quasi-subunit, SH3 and cysteine-rich domain-containing protein 3 (Stac3) [15–18] with 1:1:1:1:1 stoichiometry (Figure 2a). The α_1S_ subunit houses the channel gating and permeation mechanisms, while the accessory subunits fine-tune the biophysical properties of the channel and are involved in trafficking, membrane anchoring, and/or Ca_V_1.1-RyR organization [15–20]. Much less is known concerning the substantive roles of other junctional proteins (e.g. junctophilins) which also assist Ca_V_1.1's dual roles of L-type Ca^2+^ channel and voltage-sensor for EC coupling. This emerging topic has been recently reviewed by Perni [21].

**Figure 3.**
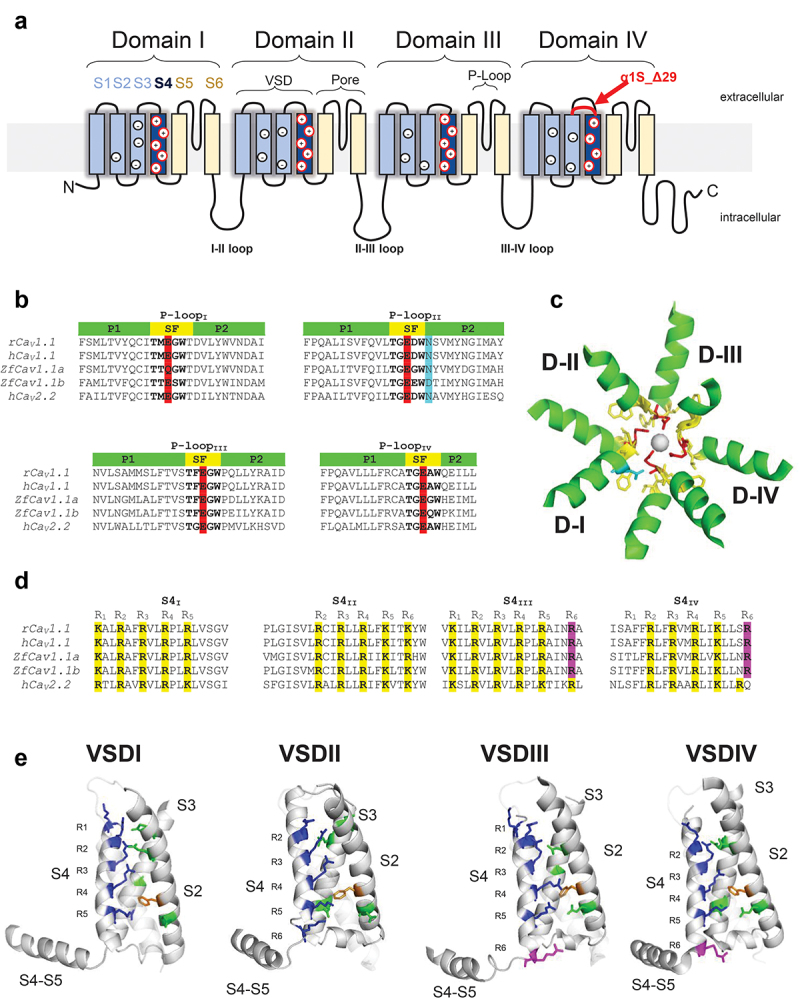
Topology of the voltage sensor and selectivity filter domain of Ca_V_1.1 α_1S_ subunit. A) Cartoon of α_1S_ subunit topology shows four homologous but non-identical domains, each containing six transmembrane helices (S1-S6). S1-S4 represents the voltage sensing domain (VSD, blue) while S6-S6 represents the pore-forming domain (Pore, yellow). Each domain contains an S5-S6 loop (P-Loop) buried in the pore acting as a selectivity filter. Intracellular loops connecting each domain are variable in length. The I–II and II–III loops are critical for EC coupling. Deletion of Exon 29 induces a 19 amino acid shortening of the S3-S4IV extracellular loop (red). Positively charged lysine and arginine within S4 are indicated as a “+” while countercharges within S2 and S3 are indicated as “-“. B) Selectivity filter amino acid sequences of rabbit, human, and zebra fish Ca_V_1.1 and human Ca_V_2.2. Critical glutamate residues used for Ca^2+^ selectivity are indicated in red, while zebra fish P-loop Ca^2+^ non-conductive mutations N617D is indicated in cyan. Selectivity filter (SF) sequence is indicated in bold and yellow, while alpha helices within P-loop are indicated in green. C) Top view of rabbit Ca_V_1.1 selectivity filter colored as in B. Alpha helices are illustrated as ribbon, while selectivity filter motif is shown as a stick. N617D mutation is indicated in blue as a stick. Two ions can bind the pore, each stabilized by P-loops from opposite domains. D) S4 helices amino acid sequences of rabbit, human, and zebra fish Ca_V_1.1 and human Ca_V_2.2. Conserved gating charges are indicated in yellow (alignment based on Ref.) [62]. Positively charged amino acids not considered as gating charges are highlighted in pink. E) Side view of rabbit Ca_V_1.1 voltage sensing domains. α-helices are illustrated as ribbons, while gating charges, countercharges, and gating charge transfer phenylalanine are illustrated as sticks (in blue, green, and Orange respectively). Note that some helices (i.e. S1) are not fully displayed to facility the visualization of other elements. Panels C and E were created with PyMol [25] from P07293. rCa_V_1.1: uniport IDs P07293; hCa_V_1.1: uniport IDs Q13698; zfCa_V_1.1a: GenBank accession no. FJ76922; zfCa_V_1.1b: GenBank accession no. AY49569; hCa_V_2.2: Q00975.

The α_1S_ subunit is a large polypeptide composed of four distinct membrane-bound repeat domains (RI, RII, RIII, and RIV) [23] (Figure 2b and Figure 3a). Each of these domains contains six membrane-spanning α-helices (S1-S6). The S1-S4 helices form the voltage-sensing module, while the S5-S6 helices constitute the channel pore (Figure 3a). Like other canonical voltage-gated ion channels, the Ca_V_1.1 quaternary structure shows four distinct voltage-sensor domains (VSDs) surrounding the pore formed by the S5 and S6 helices (Figure 2b). When viewed from an extracellular vantage point, the channel resembles a four-leaf clover, with each leaf representing one VSD (Figure 2b). The extracellular segments which link the S5-S6 helices (referred to as pore loop or P-loop) each contain a highly conserved glutamate residue (E292/E614/E1014/E1323) generating a distinct E-E-E-E motif critical for Ca^2+^ selectivity across the entire Ca_V_ channel family [24] (Figure 3b-c).

Multiple channel splice variants of Ca_V_1.1 have been identified [26,27]. Even so, only two are expressed at relevant levels, the adult Ca_V_1.1a [11] and embryonic Ca_V_1.1e [26] variants. The Ca_V_1.1a isoform originally cloned by Shosaku Numa’s laboratory in 1987 includes a 29^th^ exon which encodes 19 residues in the RIVS3-S4 extracellular linker segment, whereas the more recently cloned Ca_V_1.1e variant lacks this exon and the 19 residues that it encodes [26]. The absence of these residues in RIVS3-S4 segment confers a distinct biophysical profile to Ca_V_1.1e characterized by faster current activation, larger current amplitude, and hyperpolarizing voltage-dependence of activation relative to Ca_V_1.1a.

## Ca_V_1.1 is an L-type Ca^2+^ channel

The L-type current supported by Ca_V_1.1 has been characterized by many laboratories in various muscles from different species and conditions, but it almost certainly plays no direct role in EC coupling [28,29]. Interestingly, zebrafish and other teleosts have eliminated L-type Ca^2+^ current in muscle via specific amino acid substitution in each of their two Ca_V_1.1 coding genes (i.e. zfCACNA1Sa and zfCACNA1Sb, see Figure 3b). Consistent with observations made in mammalian systems, ablation of the Ca^2+^ conductance had no effect on the ability of either isoform to support EC coupling [30]. In particular, the N617D substitution that ablates Ca^2+^ flux resides in the RII P-loop three residues away from the extracellular glutamate that contributes to the formation of the selectivity filter (Figure 3b-c). Dayal and colleagues created a knock-in N617D mouse line for the purpose of identifying physiological processes dependent on Ca^2+^ flux via Ca_V_1.1 [8,31]. The authors found no significant differences in muscle performance or excitability when comparing N617D mice with the wild-type counterpart. Using dissociated FDB fibers from these mice, Idoux and colleagues [32] observed later that N617D conducted Mn^2+^ and Ba^2+^ in the absence of extracellular Ca^2+^. The Ba^2+^ conductance was potently blocked by Ca^2+^, indicating a higher selectivity for Ca^2+^ over Ba^2+^. On the basis of these observations and modeling, the authors concluded that the inclusion of an aspartic acid at position 617 stabilizes Ca^2+^ within the selectivity filter to the point of blocking its passage but allows lower-affinity interactions with Mn^2+^ and Ba^2+^ which permit their respective fluxes when either is the only divalent ion present. Taken together, the comparative biology approach and the subsequent generation of the N617D mouse has been quite useful in demonstrating that residues adjacent to the canonical E-E-E-E selectivity filter contribute to Ca^2+^ selectivity.

Unlike the N617D mutation, another nonconductive mutation, E1014K [9,10], disrupting the quadruple aspartate motif, contrasts with the report by Idoux et al. [32]. The E1014K mutation on Ca_V_1.1 showed enhanced muscle fatigue, altered muscle composition, and metabolic issues in an engineered mouse [9,10]. However, this mutation generates a gain-of-function in that it becomes a nonselective cation channel that conducts either Na^+^ or K^+^ depending on the membrane potential [33,34]. Thus, phenotypic defects may be related to other cation permeation within the channel rather than blockage of the Ca^2+^ conductance.

## Ca_V_1.1 voltage-sensor: Electrophysiological and molecular aspects

Understanding how ion channels detect perturbations in membrane potential has been a goal of biophysical research since Hodgkin and Huxley proposed the existence of voltage-sensing “gating particles” in the 1950s [35]. Validation of their hypothesis occurred two decades later when Martin Schneider and Knox Chandler [36] recorded non-linear capacitive currents in frog muscle fibers that activated within a voltage range comparable with contraction (between ~ −60 to +40 mV). The time integral of the outward and inward components of these recordings was nearly equivalent, satisfying the criteria of charge conservation, and bringing Schneider and Chandler to the conclusion that *“movement of charged groups […] could involve a physical motion of some tens of Angstrom units […] which could correspond to turning a molecular trigger on and off.”* We now know that these capacitive currents detected by Schneider and Chandler are macroscopic gating currents arising predominantly from the movement of voltage-sensors from Ca_V_1.1 [37–39].

The protein sequence of Ca_V_1.1 and other voltage-gated channels [11,40] revealed Hodgkin and Huxley’s “gating particles” as positively charged arginines (R) and lysines (K) present in the S4 transmembrane helices of each domain [11,40] (Figure 3a, d-e). Upon depolarization, the S4 helices translocate within the plasma membrane via an aqueous conduit formed by S1-S3 [23]. The translocation of the S4 helices causes further conformational rearrangements within Ca_V_1.1 that activate the receptor and open the channel pore. Negatively charged glutamate (E) and aspartate (D) residues present in the S2 and S3 helices interact with the positively charged, voltage-sensing residues in S4 (Figure 3a, e). A growing body of evidence suggest that these acidic “countercharges” promote a constricted interaction of the overall S1-S4, helping the creation of a hydrophobic barrier between extracellular and intracellular compartments [23,41]. Conserved phenylalanines resident in each S2 helix (i.e. the gating charge transfer center) separate the external and internal accessible crevice. Replacement of this residue by natural and unnatural amino acids can have profound impact on the voltage-dependence of current activation in K^+^ channels [42,43] and also in Ca_V_1.1 [44].

The number of arginine and lysines in each of the S4 helices of each α_1S_ domain is variable (Figure 3d and e). These residues are spaced regularly between two neutral residues that are aligned on the same face of the α-helix and are juxtaposed to countercharges in S2 and S3. Using a combination of computation and mutagenesis, Tuluc and collaborators demonstrated that VSD gating may be divided into two highly comparable groups: VSD-I and III in one group and VSD-II and IV in another [45,46]. VSD-I and III interact with two countercharges in S3 and adopt a similar charge motif “1 K4R” with lysine as the most outward charge; while VSD-II and IV interact with three counter charges, 2 in S2 and 1 in S3; with a charge motif “3R2K” and “3R1K” respectively, with lysine(s) being the most inward charge. The position of the lysine was thought to be critical since it can only interact with one countercharge, while arginine is predicted to be capable of interacting with two countercharges. The authors also sought to obtain information on current kinetics by interchanging S1-S4 from one domain to another. Their analysis suggested a more stable activated state of VSD-I and III vs.VSD-II or IV [45,47]. Additional positively charged lysine and arginine are present in the cytosolic part of the VSD-III and VSD-IV α-helices (see Figure 3d). However, these amino acids were not considered to be actual gating charges as they likely reside outside the transmembrane electrical field [41]. It is unknown for Ca_V_1.1 how far or how exactly these charged residues move in response to depolarization. While the exact motion of the different helices is still unresolved, current theory suggests that S4 outward movement generates tension into the cytosolic S4-S5 linkers, allowing pore opening and ion flow between all the S5-S6 transmembrane domains.

## Ca_V_1.1 voltage-sensor movement EC coupling domains

By and large, the voltage-sensing mechanism of other ion channels is only considered in the context of one physiological event – opening of the channel activation gate. As discussed above, the Ca_V_1.1 voltage-sensing mechanism regulates opening and triggers intracellular Ca^2+^ release from the SR. In 1973, Schneider and Chandler revealed that the non-linear capacitive current event reflecting charge movement was likely *“located in the membrane of the T-system and [charged groups] displacement constituted a step in EC coupling*.” A few years later, the simultaneous recording of Ca^2+^ transients and gating currents confirmed that charge movement preceded SR Ca^2+^ release [48]. Subsequent work confirmed Ca_V_1.1 as the voltage-sensor for EC coupling [49–51]. Much effort has been committed to investigating how conformational rearrangements within the Ca_V_1.1 heteromultimer are communicated to RyR1 across the ~10–15 nm myoplasmic gap between the T-tubule membrane and SR (Figure 1b-c), but a clear picture is still missing. The current body of evidence to date points to a cooperative mechanism involving the α_1S_ II–III loop [52], the β_1a_ carboxyl-terminus [53] and the SH3 domain(s) of Stac3 [18,54].

## Ca_V_1.1-RyR1 communication is bi-directional

Orthograde coupling at the triadic junction refers to the influence of Ca_V_1.1 over RyR1, while retrograde coupling refers to the influence of RyR1 on the open probability and activation kinetics of Ca_V_1.1 [55–57]. Genetic ablation of RyR1 decreased L-type current amplitude without a proportionate decrease in the total number of Ca_V_1.1 channels present at the plasma membrane (as measured by maximal charge movement). Moreover, pharmacologically and genetically induced changes in RyR1 conformation also exert influence on Ca_V_1.1 gating properties. With regard to the former, ryanodine treatment shifts activation of both L-type current and charge movement to more hyperpolarizing potentials [58]. For the latter, the malignant hyperthermia-linked R163C mutation in RyR1 mutation also shifts L-type current activation and charge movement to more hyperpolarizing potentials [59].

## Ca_V_1.1 Cryo-EM and in silico studies

At the beginning of the 2010s, structural information regarding Ca_V_1.1 was scarce because of the difficulty intrinsic to the crystallization of large membrane proteins and the poor resolution of single-particle cryo-electron microscopy (cryo-EM). However, breakthroughs in cryo-EM hardware and analytic tools enabled vivid channel structures with resolution below 5 Å [60]. The first Ca_V_1.1 cryo-EM structure that yielded information on the level of a single residue was produced at 4.2 Å by Ning Yan’s laboratory [61]. This watershed structure was followed in rapid succession by an even higher-resolution structure obtained at 3.6 Å [62]. Both structures included the α_1S_ subunit in complex with β_1a_, α_2_δ-_1_, and γ_1_ subunits; Stac3 was absent. In addition to providing exquisite vistas of the selectivity filter and voltage-sensing modules (Figure 2a), the structures also provided remarkably detailed information regarding intersubunit interactions. In particular, the two proteolytic fragments composing the α_2_δ-_1_ subunit (i.e. α_2_ and δ-_1_)[63]; were shown to interact in an unexpected way. The δ-_1_ subunit was found to be “inserted” into a crevice formed by α_2_[62] and was stabilized in that position by multiple disulfide and hydrogen bonds. The association of γ_1_ with α_1S_ was shown to be supported by hydrophobic residues in RIVS3 and RIVS4, RIVS4-S5 linker, and III–IV loop with the carboxyl-terminus and transmembrane segment 2 of γ_1_. Recent functional work suggests that RIVS3-4 linker interacts with γ_1_ and thus provides a plausible explanation as to why the γ_1_ subunit differentially modulates Ca_V_1.1e and Ca_V_1.1a [64]; a 19 amino acid stretch in the domain IV S3–S4 linker of α1s is absent in the embryonic splice variant Ca_V_1.1e and present in adult Ca_V_1.1a [26] (Figure 3a).

The Ca_V_1.1 cryo-EM structures also provided high-resolution of the S4 voltage-sensors [61,62]. In each repeat domain, the 4–5 basic residues responsible for sensing changes in membrane potential are aligned along one face of the S4 helix (Figure 3e). In the cryo-EM structure, in all VSDs, four of these positively charged residues (R1-R4) are located extracellular to a highly conserved phenylalanine in S2, which is thought to mark the isoelectric point of the membrane field [43]. This juxtaposition of the S4 relative to the S2 phenylalanine indicates an “active” conformation of all these VSDs as would be the case during depolarization. It is important to note that the S4 helices are of slightly different lengths, their orientations with respect to the lipid bilayer are somewhat different and neither the S4 nor S1-S3 helices are perpendicular to the membrane plane, illustrating further structural differences between VSDs.

Unfortunately, the complete structure of the 40 residues following the α_1_-interacting domain (AID) in the I–II loop and the 100 residues between repeats II–III remain uncertain. The lack of structural information regarding these segments represents a frustrating knowledge gap since both loops have been identified as being involved, if not critically important, for skeletal EC coupling [65]. However, the low resolution of these intracellular loops is not surprising since these segments are unstructured and highly mobile [66].

Through contrast and cautious extrapolation, the more recent structure of the somewhat-conserved neuronal Ca_V_2.2 channel in complex with the painkiller ziconotide has given insight into other facets of Ca_V_1.1 structure. Gao and collaborators [67] reported a Ca_V_2.2 structure with VSD-II in the “down” state while other VSDs I, III and IV are in the “up (active)” conformation. These “down” and “up” states could correspond to voltage-sensor conformations during hyperpolarization and depolarization, respectively. Sequence-specific intracellular loops and linkers of the pore-forming subunit, and the presence of the membrane lipid phosphatidylinositol 4,5-bisphosphate (PIP_2_), seemed to favor the down conformation of RIIS4 but not the S4 helices of RI, RII and RIV. VSD-II conformational differences between Ca_V_1.1 and Ca_V_2.2 might also be explained by the differences in the intracellular I–II and II–II loops that are shorter in Ca_V_2.2, and lack of RyR1 retrograde coupling as occurring for Ca_V_1.1.

The impact of PIP_2_ on Ca_V_2.2 VSD-II conformation emphasizes the effect of lipids composition on channel structure. One common concern regarding cryo-EM is a potential perturbation of channel conformation (especially voltage-sensor domains) due to protein purification in micelle detergent. Since PIP_2_ and other membrane lipids impact the gating of virtually all ion channels including Cav1.1 [68–71], a reasonable hypothesis is that the structure would likely be modified depending on membrane lipid composition. However, the first cryo-EM structure of rabbit Ca_V_1.1 in micelles was similar to the one obtained later in nanodiscs, thereby ruling out the potential conformational change due to lipids and grid preparation is the reason for these differences in the structures [72].

The Ca_V_1.1 selectivity filter vestibule and inner gate close environment were solved at high resolution when purified in either 10 mM or 0.5 mM Ca^2+^, which allowed the assignation and confirmation of two Ca^2+^ hydrated ions inside the pore [62]. A recent study determined agonist and antagonist binding within the pore in similar conditions [72,73]. Antagonists targeting specifically L-type channels like dihydropyridines (DHP), benzothiazepines (BTZ), or phenylalkylamines (PAA) have been widely used to treat hypertension and arrhythmias and experimentally to study channel function (for review see) [74]. The most recent structure of Ca_V_1.1 in complex with some sub-members of these channel blockers gave insight into the specific mechanisms by which these compounds modify ion channel permeation [72,73]. Diltiazem, verapamil and nifedipine are well-known members of the BTZ, PAA, and DHP families, respectively, while Bay-K 8644 is a DHP-like compound that can act as an agonist or antagonist depending on the stereoisomer ((+)-Bay K 8644, agonist; (-)-Bay K 8644, antagonist). While most of the structures resemble the one previously published, the orientation of RIS6 and fenestration between domains I and IV and domains II and III showed structural rearrangement when incubated with either of the drugs. All these compounds produce their effect through the pore domain or the fenestration formed by domains III and IV. The similarity in residues used to interact with the drugs could explain why some compounds could have an inhibitory effect on the binding of others, such as diltiazem and verapamil. In addition, the specificity of these drugs for L-type family members seems to be dependent on the stabilization of the compounds with polar residues within RIIIS5 and RIII S5-S6 linker are conserved amongst Ca_V_1.1–4 but not with other Ca_V_ channels.

Surprisingly, when overlapping the diltiazem- or verapamil-bound Ca_V_1.1 structures, the authors observed a structural shift in VSD-II, RIIIS5 and RIIIS6. This unexpected molecular shift suggested that the drug binding within the pore could also impact the gating mechanism. Indeed, diltiazem has been reported to impact charge movement amplitude and shift the Q-V relationship to more hyperpolarizing potentials [75,76], raising the possibility that binding of a particular compound within the pore domain may not only block the current but also impact gating and affect EC coupling.

Computation based on these structures is essential to developing selective drugs and understanding protein folding and trafficking. For example, recent work by Arrigoni and colleagues [77] based on the crystal structure of the bacterial voltage-gated sodium channel and utilizing molecular dynamics showed that in voltage-gated ion channels, including Ca_V_1.1, the VSD domains and the pore domain may fold independently from each other. As mentioned by the authors, autonomous folding of the pore domain could have implications for understanding disease-related mutations and channel trafficking.

Based on the Ca_V_1.1 cryo-EM structures and in silico modeling, Fernandez-Quintero and collaborators [47] predicted the effect of countercharge neutralization and gating charge substitution on channel gating and permeation. In addition, their molecular dynamics modeling yielded an approximation of the resting “down” conformation, energy levels and transition kinetics of the activated and resting states of VSD-I and IV. In this study, the VSD of RIV was studied for both adult and embryonic variants (VSD-IVa and VSD-IVe, respectively). With this approach, the authors estimated a motion of 15.3 Å for VSDI and 10.2 Å for VSD-IVa. Fewer ion pairs were estimated for VSD-IVa vs. VSD-Ia, a result coherent with the lower number of gating charges in RIVS4. Interestingly, the charge-countercharge pairing was different for each VSD.

The difference between VSD-IVa and IVe may be explained by a closer distance between RIVS4 and RIVS3-S2 in the embryonic variant, facilitating an R1 interaction with the countercharges. Validated with functional assays in a homologous expression system, this work confirmed the high predictive capability of modeling for Ca_V_1.1. Overall, the molecular dynamics simulations carried out by the authors show further evidence supporting the S4 movement [23,45,78,79]: α-helices turn on themselves, with different lysines or arginine interacting with acidic countercharges, generating tension in S4-S5 intracellular linker. These rearrangements cause tension on S5-S6, resulting in rotation of the S6 α-helix and opening of the channel pore.

## Distinct roles for Ca_V_1.1's VSDs

How can the Ca_V_1.1 control the slowly activating L-type Ca^2+^ current and fast SR Ca^2+^ release, each with different voltage-dependences? SR Ca^2+^ release is more immediate than the Ca^2+^ current initiation and the Ca^2+^ release from SR starts ~30 mV more hyperpolarized than Ca^2+^ current activation [80,81]. As mentioned before, Ca_V_1.1e conducts larger and faster Ca^2+^ currents with a different voltage dependence when compared with Ca_V_1.1a ^24^. These differences in gating responses for Ca^2+^ current activation and Ca^2+^ release, as well as in different Ca_V_1.1 isoforms, support the idea that each VSDs within the channel do not participate equally in either Ca^2+^ current or Ca^2+^ release. Consistent with this conclusion, Ca_V_1.1 has four similar but non-identical VSDs (Figure 3d – e). These differences in VSD structure could manifest in non-equivalent contributions to channel opening and/or RyR1 activation. Studies using mutagenesis to generate VSD Ca_V_1.1 chimeras [45,46] and more recently functional site-directed fluorometry (also known as voltage-clamp fluorometry; VCF) [82,83] support this idea.

## Functional site-directed fluorometry and Ca_V_1.1

Functional site-directed fluorometry was developed to probe the gating mechanisms of various ion channels, transporters, and receptors [84–87]. Developed in the 1990s, functional site-directed fluorometry uses cysteine-reactive conjugated dyes (often methanethiosulfonate or maleimide derivatives) to probe specific extracellularly or, in some less frequent cases, intracellularly accessible cysteines introduced via site-directed mutagenesis. When a cysteine is inserted near the extracellular part of the S4 α-helix of a voltage-gated ion channel, it can be conjugated with a cysteine-reactive dye; if the S4 moves, for example, in response to a membrane depolarization, the fluorescence emitted by the cysteine-conjugated dye changes and can be measured to track the S4 movement. These optical signals arising from S4 rearrangements are typically combined with ionic or gating current measurements using voltage clamp methods (i.e. two-electrode voltage clamp fluorometry (TEVCF) [84], cut-open oocyte Vaseline gap fluorometry (COVGF) [83,85–87], patch-clamp fluorometry) [88], or action potential fluorometry (APF) [82] via external field stimulation in parallel with measurements of action potential-induced membrane currents and Ca^2+^ transients. In this regard, functional site-directed fluorometry detects voltage-sensor motion in response to the activating stimulus (i.e. a step depolarization or an action potential). The beauty of functional site-directed fluorometry resides in that it can track the movement of individual S4 helices [89] and thereby represents a significant complement to ionic current recordings or gating current measurements, which provide indirect readouts of an ion channel’s conformational changes [89,90].

The use of functional site-directed fluorometry to investigate the biophysical properties of Ca_V_1.1 was initially problematic because of the negligible heterologous expression of the channel in non-muscle cells. Recently, functional site-directed fluorometry was utilized to track the motion of each Ca_V_1.1 S4 voltage-sensor expressed in *Xenopus* oocytes [83] and in adult murine muscle fibers [82]. In the former case, robust expression of Ca_V_1.1 α_1S_ cysteine mutants in *Xenopus* oocytes was made possible by the finding that co-expression of Stac3 facilitated the expression of Ca_V_1.1 multimers in non-muscle cells [17]. For the latter, transgenic expression of Ca_V_1.1 α_1S_ cysteine mutants in mouse muscle fibers was carried out via in vivo electroporation [91].

**Figure 4.**
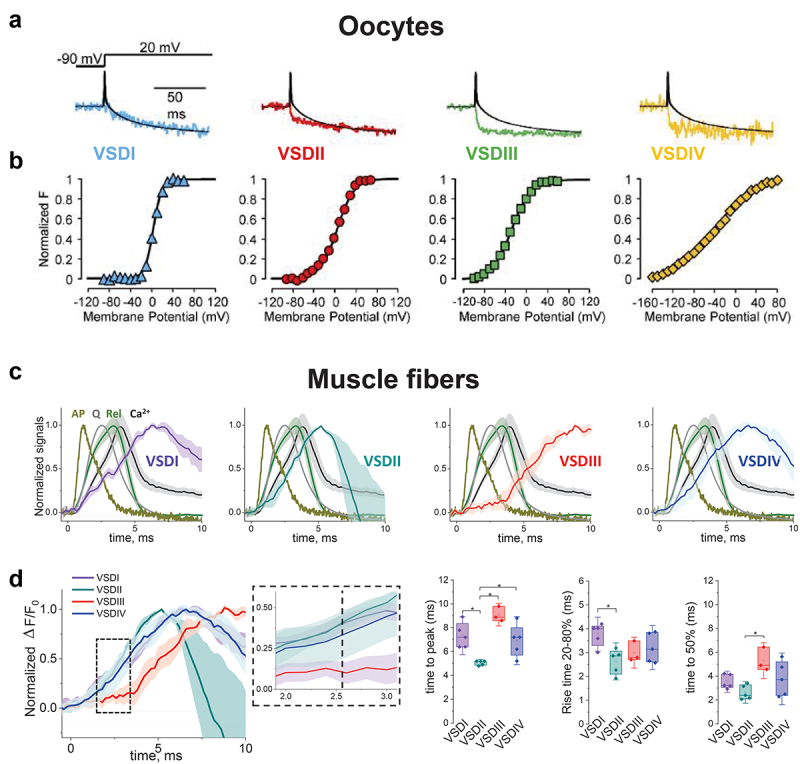
Functional site-directed fluorometry of Ca_V_1.1 S4 signals evaluated in *Xenopus* Oocytes and muscle fibers. A) Ionic current recording from cut-open oocytes (black) with 2 mM Ba^2+^ in the external and superimposed fluorometric signal from each VSDs. Note the overlap of the fluorometric and ionic signal for VSD-I. Mean voltage dependence of the fluorometric signal for each VSD from cut open oocytes voltage clamp in presence of 2 mM Ba^2+^ and fitted with a Boltzmann function. Note the differences in voltage dependence and slope of each VSDs fluorometric signal. C) Normalized fluorometric signal recorded from muscle fibers in response to self-propagated action potential by field stimulation and its comparison with optically measured membrane voltage (AP, yellow), action potential-evoked charge movement (Q, gray), Ca^2+^ transient (Ca^2+^, black), and estimated SR Ca^2+^ release flux (Rel, olive). D) Overlay of normalized fluorometric signals presented in C and kinetics quantification. Note the differences in kinetics for fluorometric signals from different VSDs. Time to peak, rise time, and time to 50% are faster for VSD-II. Panels A and B, and C, reproduced with permission from Refs. .[83] and [82], respectively. Panel D, unpublished analysis from Ref. [82]

Using a cut-open voltage-clamp paired with functional site-directed fluorometry, Savalli and colleagues [83] demonstrated that the activation kinetics of VSD-I closely followed the activation of the ionic current carried by Ca_V_1.1. A faster fluorescent signal was observed for VSD-III, VSD-IV and VSD-II (Figure 4a). Of note, a previous study from the same group reported that for Ca_V_1.2 [87], VSD-II and VSD-III (each contributing ~50% for the energy activation) followed the trajectory of the ionic current activation. While no direct analysis of the Ca^2+^ release was done in the study by Savalli and colleagues [83], the VSD-I and VSD-IV signals displayed kinetics and voltage-dependence incompatible with the Ca^2+^ release seen in muscle fibers (Figure 4a-b). They proposed Ca_V_1.1 VSD-II and/or VSD-III as the likely voltage-sensor contributor for EC coupling.

Banks et al. [82] developed a variant of the functional site-directed fluorometry in response to propagated action potentials evoked via field stimulation. Using this approach, a substantial fraction of the fluorescence signal for each VSD occurred after the time of peak Ca^2+^ release, and even more developed after the earlier peak of electrically measured charge movement during an action potential and thus could not directly reflect activation of Ca^2+^ release or charge movement, respectively (Figure 4c). This observation is in line with the idea that not all the recorded charge (total charge, Q_T_) is linked to the activation pathway of a channel (i.e. Q_p_, peripheral charge, which has no connection to gating) and only a fraction of the total charge (Q_e_, essential charge) is energetically coupled to channel function [93]. Interestingly, a sizable fraction of the fluorometric signals for VSDs-I, II and IV, but not VSD-III, overlapped the rising phase of charge moved, and even more for Ca^2+^ release (Figure 4c and d), and thus could be involved in voltage-sensor rearrangements coupled to RyR1 Ca^2+^ release.

**Figure 5.**
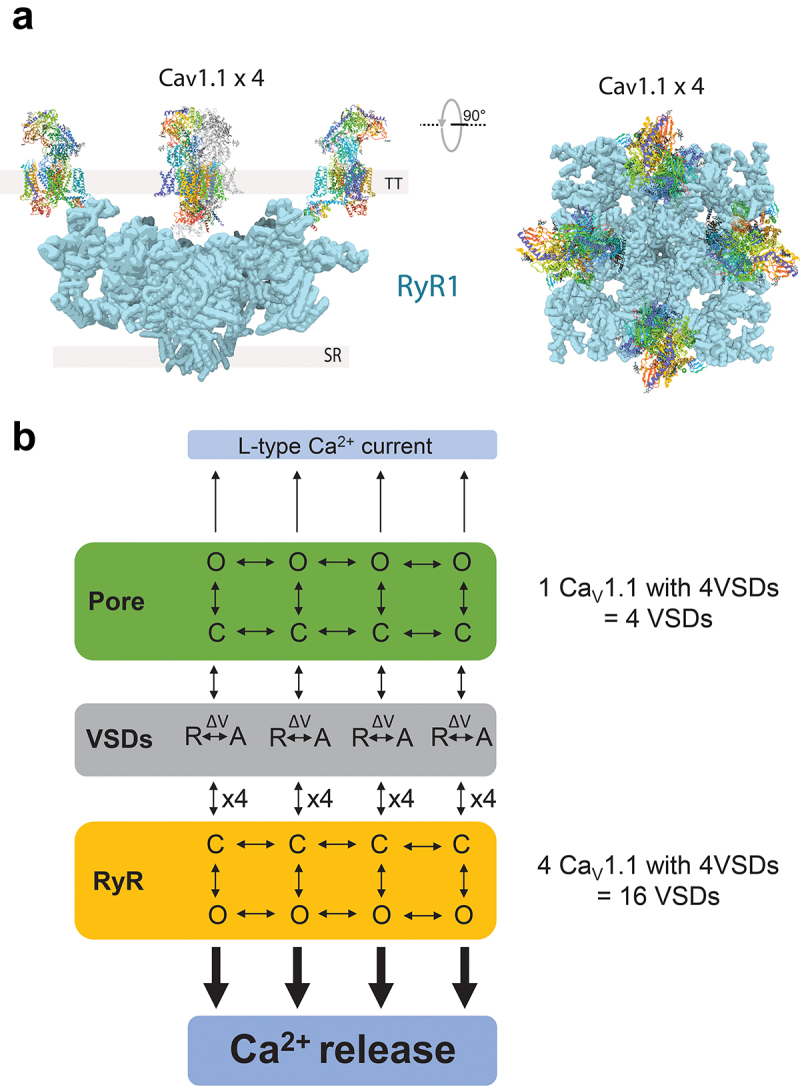
Allosteric model for L-type Ca^2+^ current and RyR Ca^2+^ release. A) Hypothetical structural representation of a Ca_V_1.1 tetrad coupled to RyR1 homotetramer. Four α_1S_ subunits (blue) are opposed to a RyR homotetramer (yellow). Ca_V_1.1 has four VSDs that alter their conformation in response to surface transmembranal voltage changes. RyR1 does not have an intrinsic voltage sensing mechanism and relay on the voltage sensing machinery of Ca_V_1.1, via mechanical coupling, to release Ca^2+^. The α_1S_-RyR1 organization depicted here is hypothetical but based on the model suggested by Samsó et al. [92]. Side and upper views (left and right respectively) in Panel A were created with BioRender and Chimera [22], PDBs: 5GJW and 5TAL for Ca_V_1.1 and RyR1, respectively. B) Allosteric scheme for voltage dependent Ca_V_1.1 channel opening and RyR1 activation. Four distinct VSDs (VSDI-IV) within one Ca_V_1.1 control Ca_V_1.1's pore conformation from close (“C”) to open (“O”) with either all or some VSDs in active (“A”) or resting (“R”) state. In parallel, four distinct Ca_V_1.1s, each with four VSDs (i.e. tetrads array) control RyR1 pore conformational change from close (“C”) to open (“O”) with either all or some VSDs in active (“A”) or resting (“R”) states. The probability of each state in VSDs (R or A) is under the influence of the membrane voltage (ΔV). Note that in principle, based on structural evidence [2,11,62], it is likely that four independently functioning Ca_V_1.1 channels (tetrads) are associated with one RyR giving four sets of four VSDs, requiring a total of 16 VSDs. However, recent fluorometric experiments [82,83] suggest some features and reconsiderations for this model: not all VSDs within Ca_V_1.1 contribute equally to gate Cav1.1 pore opening and not all VSDs contribute equally to gate RyR1 Ca^2+^ release. How many VSDs per tetrad and which of the four VSDs of Ca_V_1.1 are needed for RyR1-mediated Ca^2+^ release is unknown.

The results between the two experimental systems seem to partially disagree regarding the primary voltage-sensor(s) contribution to Ca^2+^ release by RyR1 (i.e. VSD-III vs. VSD-II). However, fundamental differences in the approach used could explain the contrast in the results (see Table 1). The elegant study by Savalli and colleagues was carried out in a heterologous system which lacked α2δ-1 and γ1. While the channel was functional under these conditions, it is well established that α_2_δ-1 and γ_1_ modulate the amplitude, kinetics and voltage-dependence of both L-type current and charge movement [64,94,95]. Each subunit likely modulates each VSD differently since each subunit interacts with a specific domain within α_1S_[87]. This concept was also elegantly illustrated by the Ricardo Olcese’s laboratory using fluorometric recordings of the four VSDs from Ca_V_1.2 and β_3_ with or without α2δ-1. The presence of α2δ-1 shifted both current activation and the fluorometric signals of VSD-I, II, and III to more hyperpolarizing potentials without affecting VSD-IV. Thus, α_2_δ-1 facilitated Ca_V_1.2 activation by increasing voltage-sensitivity of VSD-I-III, demonstrating non-identical VSDs contributions to channel opening [96]. Thus, the differences in the fluorometric signals obtained between heterologous (*Xenopus* oocytes) and native systems (murine muscle fibers) are possibly explained by the complete complement of junctional proteins present in muscle fibers but absent in the heterologous system. In particular, the absence of RyR1 and consequently, the loss of retrograde coupling, may have precipitated these different observations [55,57]. Both groups used the same cysteine modification for VSD-I, III and IV, but I was different for VSD-II. This difference in the position of the cysteine for VSDII could also impacted the trajectory of the fluorometric signal due to differential quenching of the dye [89,90]. Another potential reason for the observed differences between the outcomes of the approaches may be related to species differences between the rabbit and human Ca_V_1.1 clones expressed in muscle fibers and oocytes, respectively. However, this latter explanation appears unlikely given the high conservation of the two orthologs within the membrane-bound repeats [52].

**Table 1.** Comparison of Ca_V_1.1 fluorometric signals obtained using cut-open voltage clamp in *Xenopus* oocytes or field stimulation in mouse muscle fibers.

	Savalli et al, 2021	Banks et al, 2021
Biological system	Xenopus Oocytes	Mouse adult FDB fibers
Recording system	Cut-open voltage-clamp Photodiode	Propagated Action Potential (via field stimulation) Line scan confocal microscope
External solutions (in mM)	2 Ba(MES)2, 120 NaMES, and 10 HEPES, pH 7.0., (Supplemented by 0.5 CdCl2, 0.1 LaCl3, and 0.1 ouabain for charge movement)	L-15 media (137 NaCl, 5.7 KCl, 1.26 CaCl2, 1.8 MgCl2, pH 7.4)
Constructs	Human α1S (C133671) rabbit β1a (19,517) mouse Stac3 (Q8BZ7)	Rabbit α1S (P07293)
α1S Cysteinemutagenesis	L159C M519C V893C S1231C	L159C L522C V893C S1231C
Labeling Dye	MTS-5(6)-carboxytetramethylrhodamine [MTS-TAMRA] tetra-methylrhodamine-6-maleimide [TMRM-69]	MTS-5(6)-carboxytetramethylrhodamine [MTS-TAMRA]
Main advantages	Control of membrane voltage Fast membrane charging time Robust fluorometric signal with low background (DF/F0 ≈ 0.2–0.5%) Construct expression (4–5 Days with RNA injection) Simultaneous recording of non-linear current and fluorometric signals	Action potential depolarization Presence of all EC coupling-related proteins Native cells Ability to measure Ca^2+^ transient and fluorometric signal in same biological system
Main limitations	Heterologous environment Absence of multiple EC coupling proteins (Junctophilin, RyR, α2δ-1, etc.)	No imposed membrane voltage Fluorometric signal with high background and little amplitude (DF/F0 ≈ 0.01%) Construct expression (> 4 weeks) Co-expression of Ca_V_1.1-Cys channels with endogenous Ca_V_1.1

Despite the contrasting observations from oocytes and FDB fibers regarding the contributions of each VSD to SR Ca^2+^ release, both approaches demonstrated the ability to track VSDs translocations and confirmed the idea that the four VSDs do not contribute similarly to Ca^2+^ current and EC coupling [1,45,46,97,98]. Moreover, the elimination of VSD-I and IV as the main drivers of EC coupling is consistent with the idea that the EC coupling voltage-sensor (i.e. the *primus inter pares*) is housed in VSD-II or III and that the Ca_V_1.1-RyR1 communication conclusion travels via I–II loop (either directly [99] or through β subunit [100] or another protein) [17,101] and/or the II–III loop [52,99]. Even though the small amplitude of the fluorometric signal is currently a limitation for muscle fiber system, the advantages of both systems are complementary and may be highly synergistic when used in tandem (Table 1). Functional site-directed fluorometry holds great promise for unlocking the mysteries of EC coupling as the technology matures.

## Perspectives

It is now clear that the VSDs of Ca_V_1.1 make unequal contributions to both channel opening and EC coupling [82,83]. Recently discovered and/or more-thoroughly characterized EC coupling proteins and novel roles for old ones have been discovered. Further refinement of the structures of the Ca_V_1.1 and RyR1, ideally in their native membranes with cryo-EM and single-particle image reconstruction, is needed to identify the key determinants for Ca_V_1.1-RyR1 electromechanical coupling. New or improved existing techniques are also required to track the movement of the four Ca_V_1.1 VSDs in parallel with Ca^2+^ release measurements to obtain a complete picture of how voltage-dependent rearrangements in Ca_V_1.1 are coupled to RyR1 activation. Strategies to reveal the effective number of elemental charges per channel are needed to establish which charged residues are important for both channel gating and RyR1 coupling (see Figure 5). This new information will contribute to the generation of more evolved models which consider each VSD contribution to channel opening and RyR1 and the stoichiometry and complex allosterism of VSDs and RyR1 in the tetrads. The development of more selective drugs may prove useful in the could be added to investigation of EC coupling. Finally, because several disease-causing mutations in Ca_V_1.1 are located on VSDs, P-loop residues and intracellular loops [102,103], functional site-directed fluorometry could offer new insights into the effect of mutations that alter Ca_V_1.1 function significantly.

## Summary

The recent advancements in the synergy between structure-function analysis of voltage-sensor translocation and in silico predictions have given a new perspective on the molecular transitions which support Ca_V_1.1 channel gating, Ca^2+^ selectivity and coupling with RyR1. The voltage sensitivity, kinetic and amplitude of the S4 motions differ not only within a single Ca_V_1.1 channel but also within Ca_V_1.1 splice variants and the entire Ca_V_ family. The surrounding environment, including accessory proteins or lipidic membrane composition, also impacts channel behavior. Thus, the development of multidisciplinary approaches based on structural biology and electrophysiology will be highly useful in revealing the intricacies of Ca_V_1.1 gating. Moreover, the application of functional site-directed fluorometry in both heterologous and homologous expression systems, and native muscle fibers will be critical for testing hypotheses regarding Ca_V_1.1 transitions that are essential for EC coupling.

## Data sharing statement

All journal articles cited in this work are listed on PubMed (https://pubmed.ncbi.nlm.nih.gov), except for reference 44, which can be found on the publisher’s website.
